# Understanding ethical challenges of family planning interventions in sub–Saharan Africa: a scoping review

**DOI:** 10.3389/fgwh.2023.1149632

**Published:** 2023-08-22

**Authors:** Eloisa Montt-Maray, Lamiah Adamjee, Nour Horanieh, Alice Witt, Thaïs González-Capella, Anja Zinke-Allmang, Beniamino Cislaghi

**Affiliations:** ^1^ Department of Global Health and Development, London School of Hygiene and Tropical Medicine, London, United Kingdom; ^2^ Department of Epidemiology, Biostatistics and Occupational Health, McGill University, Montreal, QC, Canada; ^3^ Department of Family and Community Medicine, College of Medicine, King Saud University, Riyadh, Saudi Arabia

**Keywords:** family planning interventions, ethics, contraception, sub-Saharan Africa, reproductive health

## Abstract

**Background:**

Improving the design of family planning (FP) interventions is essential to advancing gender equality, maternal health outcomes, and reproductive autonomy for both men and women. While progress has been made towards applying a rights-based approach to FP interventions in sub-Saharan Africa, **t**he ethical implications of FP interventions has been underreported and underexplored. Several ethical challenges persist related to measuring success, choice, and target population.

**Methods:**

We conducted a scoping review to understand if and how FP interventions published between 2000 and 2020 within sub-Saharan Africa address the ethical challenges raised within the literature. We identified a total of 1,652 papers, of which 40 were included in the review.

**Results:**

Our review demonstrated that the majority of family planning interventions in sub-Saharan Africa place a strong emphasis, on measuring success through quantitative indicators such as uptake of modern contraception methods among women, specifically those that are married and visiting healthcare centres. They also tend to bias the provision of family planning by promoting long-acting reversible contraception over other forms of contraception methods potentially undermining individuals' autonomy and choice. The interventions in our review also found most interventions exclusively target women, not recognising the importance of gender norms and social networks on women's choice in using contraception and the need for more equitable FP services.

**Conclusion:**

The results of this review highlight how FP interventions measured success through quantitative indicators that focus on uptake of modern contraception methods among women. Utilising these measures makes it difficult to break away from the legacy of FP as a tool for population control as they limit the ability to incorporate autonomy, choice, and rights. Our results are meant to encourage members of the global family planning community to think critically about the ethical implications of their existing interventions and how they may be improved. More public health and policy research is required to assess the effect of applying the new indicators with the FP community as well as explicitly outlining monitoring and evaluation strategies for new interventions to allow for programme improvement and the dissemination of lessons learned.

## 1. Introduction

The contentious ethical history of family planning (FP) interventions delivered between the 1950s and 1994 have been well explored in scholarly enquiry (1–3). Although initial interventions were designed to address population growth issues, critics started to raise ethical questions about interventions that did not place women’s autonomy at their centre. The 1994 international Conference on Population in Cairo marked a significant milestone in the discourse surrounding ethical considerations in FP, with the international community committing to adopt a rights-based and women centred approach to programming that would put the users' reproductive and contraceptive choices first. Almost twenty years later at the 2012 London Summit, a new partnership (FP2020) was established with the goal of reaching 120 million new contraceptive users by 2020 while committing to a rights-based and women-centred approach (4, 5). The next iteration of the partnership, FP2030, was rolled out in 2021 with an emphasis on voluntarism, choice and autonomy within FP interventions (6).

FP interventions can take many different forms, including providing contraceptive methods, counselling, mass media campaigns, community-based distribution, and integration with other health services (7). The intention of these interventions can be broadly placed into two groups: meeting unmet needs and generating demand for FP (8, 9). In this review, we refer to interventions aimed at fulfilling each of these objectives as “FP interventions”. Since 1994, there has been limited critical enquiry into whether and how ethical issues might persist within FP interventions. Scholars have identified several areas where ethical challenges must be taken into consideration and reflected upon in FP interventions, such as, the range and type of methods that they offer (7, 10); the population that they target (11, 12); and the measurements used to articulate their success (13). As it was the case before 1994, these ethical considerations relate to the way in which FP interventions were largely based on whether they fulfilled the reproductive and contraceptives needs of their clients. Despite the literature surrounding these ethical considerations, FP interventions are still designed in ways that may or may not meet the desires and needs of their intended beneficiaries.

The first ethical challenge concerns “methods mix,” which refers to the variety and type of contraceptive methods an intervention (13–16). WHO guidelines and supporting literature have outlined that offering women and couples a variety of contraceptives options with information is critical to ensure persons can exercise choice, receive high quality FP services, and supports the adaptability of FP strategies across diverse communities (14, 17). To promote autonomy throughout the decision-making process, FP interventions must make sure that people have access to enough information and contraceptive options to choose the one that best meets their needs. Despite efforts made by governments and organisations to increase contraceptive method options, critics have questioned whether existing interventions indeed provide women with appropriate options and information to make a choice (13, 15, 16, 18, 19). More specifically, it has been indicated that in some low resource regions, the promotion and marketing of a certain contraceptive methods such as long-acting reversible contraceptive (LARC) has been favoured and the variety of options made available are limited (16, 20, 21). This can lend to a narrow focus on contraceptive use as a means to fulfil intervention targets, rather than a focus on establishing true user preference which meets the unique reproductive health needs and desires of individuals (13). Interventions that respect and support women's choices should offer a wide range of options for contraceptive methods (described sometimes as “contraceptive method mix”) to enable women and couples to conduct their own risk benefit assessments. Moreover, integrating refusal, safe abortion, and fertility enhancement into the framework of contraceptive information have been considered as imperative to comprehensive contraceptive services (13, 15, 18, 22, 23).

A second ethical challenge relates to the population that FP interventions target. To improve reproductive health outcomes and overall wellbeing for people and communities, inclusive FP interventions are important as they enable people of all genders and circumstances to make informed reproductive decisions. Despite growing interest in the need to make FP more inclusive of subgroups such as men, adolescents, and unmarried women (12, 24–26), most FP interventions are designed under the assumption that women should make the majority of decisions regarding the use of contraception (27–29). Consequently, FP interventions typically target women who use contraception and overlook others involved in the FP decision-making process, which is exacerbated by cultural and societal norms (30). Recent literature has denoted that men and boys are key drivers or inhibitors of FP and can be safely incorporated into FP programs in ways that support gender equality and result in positive maternal and child health outcomes (12, 31). Local norms that unmarried women should not engage in sexual activity and should thus not have access to FP have similarly resulted in their exclusion from FP interventions (28, 29, 32). There is also ample evidence that adolescents lack access to FP services for numerous reasons including gender and local norms despite the recognized notion that increasing adolescent engagement in FP has positive impacts across the lifespan (33–36). Better understanding of the populations that treatments are intended to reach is necessary to unpack this ethical challenge.

Measures of success are imperative to evaluate the impact of an intervention, mark progress, and provide the groundwork for evidence-based decision making (37). In the context of FP, although efforts have been made to apply a reproductive rights-based framework to interventions, the overall focus on reducing fertility and population growth continues to permeate. As a result, the indicators utilized to measure the effectiveness of interventions has been closely associated with increasing contraceptive use as a way to lower fertility and population growth (38). This brings us to the third ethical issue where critics have challenged the indicators employed to measure the success of interventions, particularly measurements of contraceptive prevalence (13) and unmet need (13, 39), designed to monitor population level demographic changes (38). Senderowicz (13) argues that the measurement of contraceptive prevalence fails to capture the degree of reproductive autonomy, disregarding if the client wanted to use contraceptive methods even if they have not expressed a desire to (8, 13, 37, 40–43). Similar target-oriented measures might unwittingly result in programmatic practices that focus more on encouraging women's uptake (to meet interventions goals) than helping them make informed choices regarding whether to use modern contraceptive methods or not, and which method to use. Additional indicators that incorporate women's agency in family planning have been proposed by scholars, however, limited uptake in SSA has been cited due to the data requirements and knowledge gap between researchers and intervention implementers (23, 44–49).

Today, a rights-based approach has been prominent in FP2020 and new FP2030 initiatives specific to measuring unmet needs. Women with unmet need for FP are “fecund and sexually active but are not using any method of contraception, and report not wanting any more children or wanting to delay the birth of their next child” (50). Critics have highlighted that rather than measuring intention to use a contraceptive method, the unmet need indicator rests on the assumption that all women who do not wish to become pregnant, want to use a modern method of contraception, even if they have not expressed to do so (40, 51, 52). It has also been identified that this indicator, originating from scholars in the Global North in the 1960s to help governments forecast aggregate-level estimates of fertility reduction in relation to national shifts in contraceptive prevalence, is ill-suited for understanding the reproductive needs of individuals (13, 40). Furthermore, men's unmet need for FP is not captured by the standard indicator “because it is impractical to ask men infecundity questions” (43), meaning that a large portion of a target population is not captured in formative needs estimates (43). Within the unmet need strategy, a recent wave of international FP action included programmatic strategies to, specifically, generate demand for modern contraceptive methods. These “demand generation” interventions aim to affect women's contraceptive practices by increasing uptake of modern contraception methods (23–25).

We conducted this scoping review to understand how the design of FP interventions corresponds with each of the ethical challenges outlined in the literature since the international community adopted a women-centred and right-based approach. We reviewed studies describing interventions that took place between 2000 and 2020 in sub-Saharan Africa (SSA). We chose to study this region as it includes high priority countries for international FP initiatives (53), contraceptive use varies greatly within the region (54), and there are distinct ethical considerations relevant to the interplay of social and gender norms, fear of infertility, stigma, and financial constraints (54). Furthermore, there are substantial gaps in the literature on the ethics of family planning in SSA and it has not yet been determined how these specific ethical challenges in FP interventions affect a broader notion of contraceptive autonomy (32, 54, 55). Several studies call for more qualitative and longitudinal studies to broaden our comprehension of the complex interplay between different individual and community factors that influence women's contraceptive behaviour and how these dynamics change across the different phases of their lives in SSA (13).

This paper will present the results of our review covering three key areas: (1) How FP interventions were designed and implemented; (2) Which population FP interventions targeted; and (3) Which outcomes were used to measure success of FP interventions. No review to date has described FP interventions implemented in SSA through the lens of these ethical challenges. Instead, most reviews report on the effectiveness of family planning interventions and meeting quantitative goals. Our scoping review aims to synthesize existing literature to advance the understanding of ethical considerations in FP interventions. Moreover, this review aims to provide organisations designing interventions with, important insights into elements that can be incorporated into culturally appropriate family planning programs to respect people's agency and voice.

## 2. Methods

We conducted this scoping review following the Preferred Reporting Items for Systematic Review and Meta-Analysis extension for Scoping Reviews (PRISMA-ScR) guidelines (33). Our main objective was to explore how each of the ethical challenges raised in the literature have been addressed by the design of FP strategies. Therefore, we based our methodology on the formal guidance for considering scoping reviews produced in 2015 by methodological working group of the JBI (55, 56).

### 2.1. Search strategy and inclusion criteria

The search strategy was an iterative process, which resulted in modifications to the initial search strategy. We first searched for demand generation FP interventions implemented in SSA, including mass media (e.g., radio, television, soap opera and drama), interpersonal communication (e.g., community-and facility-based interventions one-on-one discussions and small-group sessions) and cost-mitigation interventions (e.g., vouchers or cash transfers to reduce cost of contraceptives for users) (7). Our initial search returned few articles focusing solely on FP demand generation interventions within SSA, thus we expanded our search to include interventions addressing unmet needs in SSA. As interventions and programmes that address unmet need include programmatic strategies to generate demand for modern contraceptive methods, we refer to both demand generation and unmet need approaches as “FP interventions” throughout this review. Included studies were: (1) quantitative, qualitative or mixed-method designs published in peer-reviewed journals; (2) written in English; (3) published between January 1, 2000, and October 1, 2020 to capture the period a few years after the FP community's decision to adopt a rights-based approach (1994) and the end of the FP2020 partnership (2020); and (4) about FP interventions implemented in SSA. For studies that may have been missed in the original database search, we reviewed the reference list of included studies and located relevant articles. Studies were excluded if they focused on interventions in health services more broadly, by which FP was a smaller component of the intervention, migrant populations or asylum seekers from SSA in other countries, or did not meet the inclusion criteria. Reviews, commentaries, and study protocols were also excluded.

**Figure 1:**
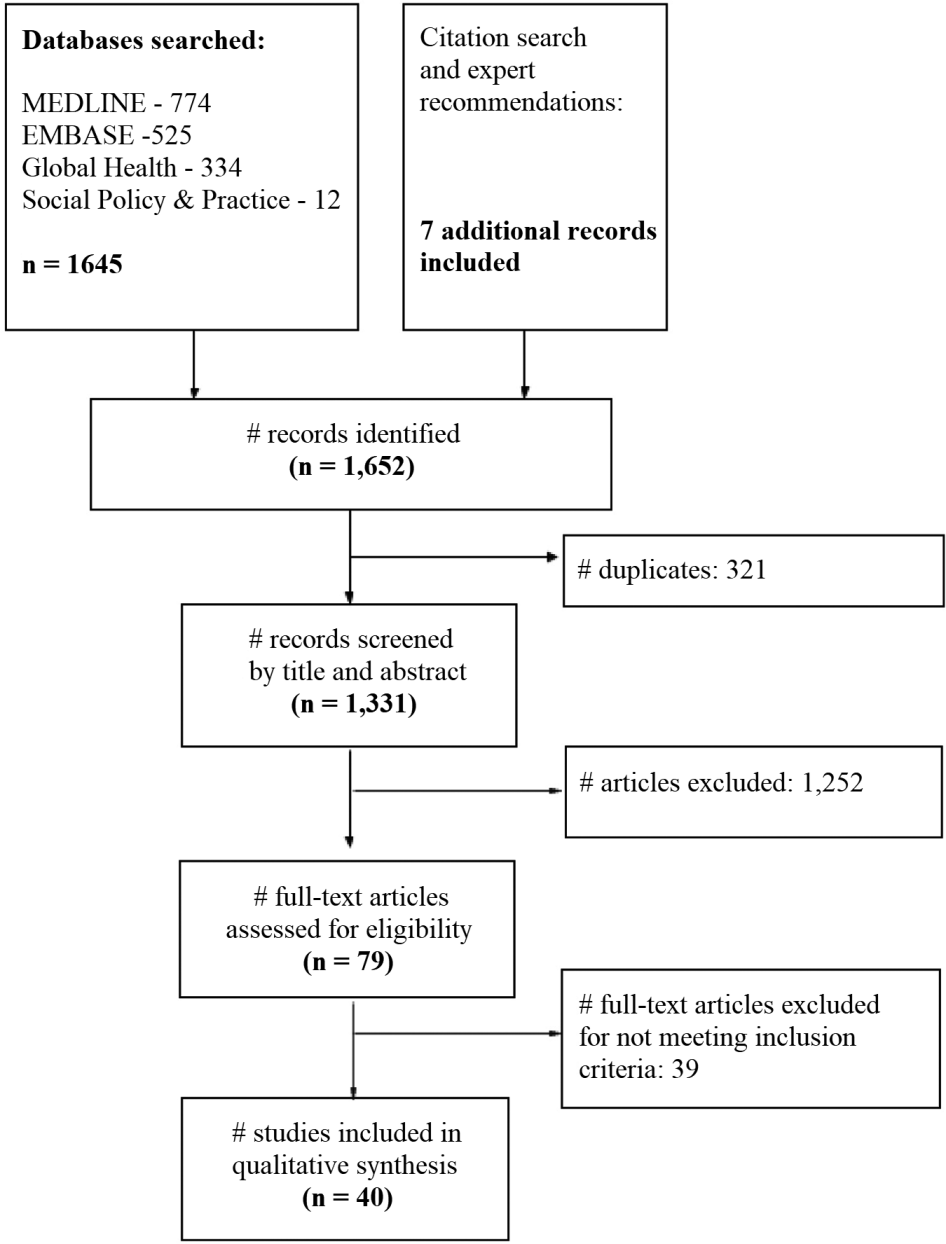
PRISMA flowchart.

We selected four relevant databases for this scoping review: MEDLINE, EMBASE, Global Health, and Social Policy & Practice. Two researchers searched the selected databases using the following combination of terms: “contraception” OR “family planning”; AND “unmet need” OR “demand generation”; AND “Africa” OR the list of countries classified as SSA by the World Bank, using truncation but without including demonyms to avoid studies about migrant population in other settings. Additionally, MeSH terms, when available, were also included for each database to complement keywords, such as “Africa south of the Sahara” or “contraception behaviours”. The search strategy was consistent across all databases. We conducted the search strategy on October 1, 2020, and updated it on May 3, 2023. We identified a total of 1,331 studies of which 321 duplicates were removed. The titles and abstracts of the remaining 1,331 studies were evaluated based on the inclusion criteria by three authors (LA, EM, and TG). Seventy-nine (*n = *79) papers met the inclusion criteria and were divided between five authors (EM, LA, AW, TG, NH) to complete the full-text review. Discrepancies were discussed until a consensus was reached. Following the full-text review, a further 39 papers were excluded leaving a total of 40 papers for data extraction (see Figure 1—PRISMA flowchart).

We extracted the following data from the selected articles: authors, title, year, country, study type, funding source, description of the intervention, type of intervention, outcome indicator, outcome indicator type, and target population (See Supplementary Table S1—Summary of included studies).

## 3. Results

Of the 40 studies that were included in this scoping review (See Supplementary Table S1), 70% (*n* = 28) were quantitative, 12% (*n* = 5) were qualitative, and 18% (*n* = 7) were mixed methods.

Among the FP interventions, 35% were implemented in Eastern Africa (*n* = 14), 28% in Western Africa (*n* = 11), 11% in Central Africa (*n* = 4), 13% in Southern Africa (*n* = 5), and the remaining 15% (*n* = 6) across multiple countries within SSA.

The interventions were funded by a variety of donors, but half (*n* = 28) were financed by US organisations alone: 14 were supported (entirely or in part) by USAID (57–70), six by the Bill and Melinda Gates Foundation (BMGF) (71–76), and two by university departments (77, 78). The following describes the allocation of financial resources towards the research; however, the authors did not specifically mention the funding sources for the interventions being studied. The subsequent US based organisations funded at least one study: International Rescue Committee (79), National Institute for Health (80), the Society of Family Planning (81), Planned Parenthood Global (82), Engender Health and Tides Foundation (83), and CARE (84). Two studies were funded by European organisations (65, 85) and two studies received funding from multilateral organisations, including UNDP, UNFPA, UNICEF, WHO and World Bank (32, 86). One study received funding from multiple organisations and university departments (87) and another study received funding from the Ethiopian government (88). Finally, two studies reported anonymous funding (89, 90), and four did not include a funding statement (91–94).

### 3.1. Intervention design

Of the papers we reviewed, 47% (*n* = 19) described supply-side interventions (i.e., interventions that aim to improve FP access and service-delivery), 15% (*n* = 6) described demand-side interventions (i.e., activities aiming to increase the acceptability and use of contraceptive methods), and 38% described interventions that were a combination of both (*n* = 15).

#### 3.1.1. Supply-side interventions

Supply-side interventions aim to improve access to modern contraceptive methods, provide high-quality FP services, prevent stock-outs, give evidence-based training to service providers and counselling on contraceptive methods to clients (7). The reviewed studies described three approaches: (1) Supplying contraceptive methods (including abortion and post-abortion care and voluntary sterilisation) (*n* = 19); (2) Providing counselling to users (*n* = 13); and (3) Training service providers on provision of modern contraceptive methods (*n* = 12).

##### 3.1.1.1. Supplying contraceptive methods

All 19 studies describing interventions that supplied contraceptive methods explicitly reported the contraceptive methods offered (58, 59, 62, 67, 69, 70, 72, 75, 79, 80, 83, 84, 86, 87, 89–93). Seventeen studies described interventions providing contraceptive method mixing approaches (58, 59, 62, 69, 70, 72, 75, 76, 79, 83, 84, 86, 89–93). Of these, eight did not describe which methods were offered (58, 72, 75, 76, 79, 84, 90, 93). One study offered three types of FP methods (86), while eight other studies offered four or more methods (59, 62, 69, 70, 83, 89, 91, 92). Eight of the 19 studies described interventions that invested considerable effort, time, and/or resources into prompting users to adopt long-acting reversible contraceptive methods (LARCs) over other methods (59, 67, 69, 79, 84, 90–92). Two focused only on provision of LARCs (67, 80), while one study described an intervention which exclusively provided injectables (87). Three interventions included voluntary sterilisation as a contraceptive method (83, 86, 95). The rationale provided for investing in LARCs was that they guarantee lower failure rates compared with short-acting methods (59, 62, 69, 79, 86, 90, 92) or the need to expand contraceptive choice since there is lack of availability or knowledge of LARCs (69, 70). No interventions included fertility treatment as part of tackling unmet need for FP services. Two interventions included abortion or post-abortion care as a FP service (79, 90).

##### 3.1.1.2. Providing counselling to users

Fourteen studies described interventions which provided users with FP counselling. Out of these, 12 interventions counselled women who were visiting sexual and reproductive health (SRH) services and exclusively provided information on biomedical contraceptive methods (32, 57, 62, 65, 72, 75, 78, 79, 81, 87, 89, 96), while one study did not specify what short-term method or modern methods were provided (i.e., if these included condoms) (70, 76). Likewise, one study described how an intervention offered information about “traditional” contraceptive methods, but did not elaborate further (89). It was common for counselling to be focused on promoting LARCs and three studies described interventions which provided postpartum women with counselling specifically about LARCs (78, 86, 89). One study described an intervention that used mobile phone technology for FP counselling (78). Three studies explicitly mentioned including men in the counselling process, however only as part of couples rather than as individuals (65, 76, 81). Only one study focused on youth (70).

##### 3.1.1.3. Training service providers

Twelve studies described interventions that trained service providers in contraceptive method provision. These trainings provided information on the range of contraceptive methods available, FP counselling techniques, and clinical procedures for fitting methods such as intrauterine devices (IUDs) and implants (60, 63, 66, 71, 74, 79, 82, 85, 86, 89, 90, 96). Eight studies described interventions which offered training on LARC insertion and removal (60, 71, 74, 82, 86, 89, 90, 96). Of these, three offered training only on LARCs and five offered training on both LARCs and other methods. Two studies described interventions that did not include any training on LARCs and focused only on short-term contraceptive methods (63) and fertility (66).

Two studies described interventions that included exercises to encourage providers to be self-reflective about their influence on a client's FP decision-making (82, 89). One intervention encouraged service providers to share their opinions on postpartum IUDs with trainers who offered them feedback on possible prejudices they held (89), while the other encouraged providers to give non-judgmental and respectful counselling regardless of a client's background or sociodemographic characteristics (82).

#### 3.1.2. Demand-side interventions

Interventions that aim to increase awareness as well as acceptability and use of contraception are often referred to as demand-side interventions (7). These types of intervention are heterogeneous and sometimes combine demand- and supply-side interventions (7). However, for the purpose of this review, we describe three demand-side approaches: (1) mass media campaigns (*n* = 10); (2) interpersonal communication activities (*n* = 15); and (3) financial support (*n* = 7).

##### 3.1.2.1 Mass media campaigns

Ten studies described interventions that used mass media campaigns to promote the uptake of FP methods through radio programmes, television shows or commercials, and print media (59, 62, 64, 71–76, 93). No mass media interventions incorporated social media components, but two interacted with potential users and/or couples via SMS (70, 81).

##### 3.1.2.2 Interpersonal communications

Sixteen studies described interventions that used interpersonal communication activities (62, 64, 70–75, 77, 79, 82, 88, 90, 91, 93, 97). Nine of these interventions targeted whole communities, either *via* community group discussions (with women, men, community stakeholders or religious leaders) or through community events (such as football matches, campaigns in markets or concerts) (43, 62, 73–76, 79, 91, 93). Three interventions included a peer-male outreach component and seven studies described interventions which provided financial support (82, 88, 97).

##### 3.1.2.3 Financial support

Four interventions used voucher schemes (59, 69, 70, 91), six subsidised the cost of contraceptives (62, 72, 75, 82, 87, 91), and three subsidised the cost of attending an FP service (62, 72, 75). Notably, only one intervention aimed to increase uptake of injectables by providing cash incentives to community health workers, who were paid for each injectable they administered while subsidising the cost of injectables (but not other methods) for clients (87).

### 3.2. Target populations of FP interventions

More than half (*n* = 22) of the studies reviewed described interventions that exclusively targeted women (58, 59, 60, 62, 63, 68, 69, 71, 72, 75, 78, 82–84, 86, 87, 89–94) (see Table 1). Most studies described interventions that targeted women of reproductive age and women seeking other services. For example, Cooper et al. (58) described an intervention that targeted postpartum mothers who brought infants to the hospital for routine immunisation, where the intervention provided them with information about FP and same-day referrals to FP services. Six interventions only targeted specific sub-groups of these, including mothers accessing maternal healthcare services after the postpartum period and women seeking non-birth-related sexual health services, like genital fistula repair (58, 60, 78, 83, 86, 90). Interventions that targeted postpartum or post-abortion women emphasised the need to prevent unintended pregnancies in these groups and ensure healthy birth spacing “during a particularly vulnerable time for women” (60) (58, 78, 86, 90). Two studies described interventions that exclusively targeted men (73, 97) and seven described interventions that targeted heterosexual couples (64, 65, 67, 79, 80, 81, 97).

**Table 1:** FP intervention target populations.

Target population	Intervention
Adult women exclusively	Akamike et al. (94)
	Babalola et al. (93)
	Babalola et al. (68)
	Bellows et al. (69)
	Benfield et al. (83)
	Cooper et al. (58)
	Dev et al. (78)
	Duvall et al. (59)
	Eluwa et al. (60)
	Ezugwu et al. (92)
	Gold et al. (62)
	Keogh et al. (32)
	Kiemtoré et al. (82)
	Lemani et al. (71)
	Ngo et al. (91)
	Rattan et al. (84)
	Samuel et al. (90)
	Speizer et al. (72)
	Tran et al. (86)
	Tumlinson et al. (75)
	Weidert et al. (87)
Adult men exclusively	Okigbo et al. (73)
Adult men and women community members	Aristide et al. (77)
	Krenn et al. (76)
Youth	Burke et al. (70)
Health providers exclusively	Graffy et al. (85)
Health providers and women	Assaf et al. (57)
	Hackett et al. (89)
Community-based workers and women	Hoke et al. (63)
Heterosexual couples exclusively	Harrington et al. (81)
	Ho and Wheeler (79)
	Igras et al. (64)
	Malama et al. (65)
	Mukamuyango et al. (80)
	Shattuck et al. (97)
	Thurston et al. (67)
Multiple target populations (more than two)	Eva et al. (96)
	Ojanduru et al. (66)
	Sedlander et al. (88)
	Tang (74)

While most interventions targeted women individually, two studies described interventions that also targeted members of women's social networks. Ojanduru et al. (66) described an intervention targeting community leaders and youth facilitators (66), while Sedlander et al. (88) described an intervention targeting men in the community, adolescents, teachers, health extension workers, and religious leaders (88). The authors of these two studies proposed that social networks reduced social pressure on women to be the sole decision makers about FP by incorporating other decision makers that could support women in their decision making process. Similarly, three studies simultaneously targeted women, healthcare providers and community health workers (63, 78, 89). It was noted in two studies that the inclusion of community health workers allowed interventions to reach underserved remote areas and including healthcare providers provided an opportunity to reach women while they were engaged in care (63, 78).

### 3.3. FP intervention outcome indicators

The FP interventions measured success using a range of outcome indicators including modern contraceptive uptake, number of participants reached, user acceptability, knowledge of modern contraception among FP users and providers; and contraceptive method mix used by clients (see Table 2).

**Table 2:** Primary outcome indicators for interventions reviewed.

Primary outcome indicator	Intervention
Modern contraceptive uptake or use	Akamike et al. (94)
	Babalola et al. (93)
	Babalola et al. (68)
	Bellows et al. (69)
	Cooper et al. (58)
	Duvall et al. (59)
	Eluwa et al. (60)
	Harrington et al. (81)
	Ho and Wheeler (79)
	Kiemtoré et al. (82)
	Krenn et al. (76)
	Mukamuyango et al. (80)
	Ngo et al. (91)
	Okigbo et al. (73)
	Rattan et al. (84)
	Samuel et al. (90)
	Sedlander et al. (88)
	Shattuck et al. (97)
	Speizer et al. (72)
	Tang (74)
	Tran et al. (86)
	Tumlinson et al. (75)
	Weidert et al. (87)
Cost per couple-year of protection (CYP)	Gold et al. (62)
	Lemani et al. (71)
	Thurston et al. (67)
Number of counsellors receiving training in FP methods	Malama et al. (65)
The competency of Community-Based distribution (CBD) workers to provide DMPA services safely and correctly	Hoke et al. (63)
Changes in FP knowledge and confidence in delivering FP counselling	Graffy et al. (85)
User acceptability of intervention	Babalola et al. (68)
	Dev et al. (78)
	Igras et al. (64)
	Krenn et al. (76)
Number of participants the intervention reached	Ojanduru et al. (66)
Client satisfaction with FP services	Assaf et al. (57)
Knowledge and willingness to accept post-partum IUDs	Ezugwu et al. (92)
Experiences with and attitudes toward IUD	Eva et al. (96)
Patterns and determinants of contraceptive use	Aristide et al. (77)
	Keogh et al. (32)
Perspectives of providers on implementation, and receptiveness of women toward postpartum IUD services	Hackett et al. (89)
Changes in contraception knowledge and use	Benfield et al. (83)

Twenty-three interventions used modern contraceptive uptake as their primary outcome indicator (58–60, 68, 70, 72–76, 79–82, 84, 86–88, 90, 91, 93, 94, 97). Additional quantitative outcome indicators used as measures of success in interventions included the number of participants the intervention reached (66) and the number of counsellors who received training in FP methods (65).

The studies reviewed generally did not include measurements of contraceptive method mix used by clients, however, one study adopted this measure as a secondary indicator of success within the intervention (79).

Although almost all papers applied quantitative indicators to assess success, five studies used qualitative methods to understand clients' experiences of and attitudes towards FP interventions (32, 77, 84, 89, 96). For example, one study examined perceptions of the FP services available in the community, modern contraceptive use (such as percentage of participants who approved of FP after the intervention), and percentage of participants who discussed FP with a spouse or partner after the intervention (93). Another study used experiences of and attitudes toward IUDs as an outcome indicator (96). Three studies described interventions which measured the acceptability of FP services to users, community members, and FP providers and their satisfaction with the service received (57, 64, 78).

FP knowledge was integrated as a measure to understand the quality of counselling services in three studies (83, 85, 92). For instance, one study described an intervention which, upon providing a wide range of method choices to women in antenatal units, evaluated women's knowledge of and willingness to accept postpartum intrauterine devices. Two studies described interventions which captured changes in contraceptive knowledge and use before and after implementation, highlighting the use of paired measures instead of individual measures (66, 83). One study described an intervention which measured changes in FP knowledge and confidence in delivering FP counselling among health providers specifically (85).

## 4. Discussion

This scoping review identified 40 studies describing FP interventions implemented in SSA between 2000 and 2020. We investigated three research questions related to: (1) the design and types of FP interventions (2) the populations targeted by FP interventions; and (3) the indicators used to measure FP intervention success. We found that FP interventions included in our study mostly use indicators focused on measuring uptake of modern contraception method use. We also found that among interventions that supplied contraceptives, a notable number of interventions focused on promoting LARCs with limited provision of contraceptive method mix, and that most interventions exclusively targeted women.

The discourse on decolonising global health has highlighted that the international development—and family planning—agendas are largely developed by institutions based in the Global North. The FP agenda has many organisations and funding agencies based in the Global North that set global targets, choosing the preferred indicators of success, and funding most FP interventions both in the Global North and Global South (98, 99).

Critics have argued that the agenda set by key players in the family planning community may not be able to fully meet the desires of individual women. When the success of FP interventions is primarily measured through contraceptive uptake and use, outcomes such as informed refusal of contraception, abortion, or expressed desires for increased fertility are not considered legitimate intervention outcomes (23, 100). These indicators reflect the targets and goals of a subset of the international FP community, such as the FP2020 goal to increase the number of contraceptive users by 120 million by 2020 (101). However, the exclusive focus on contraceptive uptake by some important FP players may have negative implications on women's ability to make informed decisions about FP use since practitioners can be (more or less overtly) pressured to reach specific uptake targets rather than the preferences of individual clients (102).

Further, some scholars have suggested that the widespread use of numerical indicators such as modern contraceptive prevalence rate (mCPR) represents a neo-colonial imposition on fertility that assumes all women want to have smaller families and achieve those small families through use of a contraceptive method, which may not be the case in diverse context, such as SSA (13, 40). In fact, studies have reported that women lack autonomy in choosing to use family planning services as well as the preferred method of choice (103, 104, 105). Many of the numerical indicators that focus on measuring uptake lack the ability to capture informed choice and autonomy. Without the proper tools to measure autonomy and choice, family planning services may not be applying a rights-based approach. Although measuring autonomy and choice is a difficult task, several funding organisations have called for the need to measure such variables and scholars have proposed novel measurement tools that can be applied in such programmes (13, 106, 107).

We also found that almost all interventions focused on promoting modern contraceptive methods (such as LARCs) and training health practitioners on providing them. The tendency to prioritise LARCs over short-acting and traditional methods has been reported in the wider literature and has been often justified based on the high efficacy of LARCs (18, 20, 108). A rights-based approach, however, demands that priority be given to people's desires, not to what is most effective; clients might still want to use what is most effective, but they should be given the choice to choose between a mix of methods. In particular, the issue of method mix within rights-based FP interventions has important implications in resource-poor settings, not least because weak supply chains can cause reduced availability of multiple forms of modern contraception and stock-outs (22, 109). Furthermore, as LARCs require medical assistance for insertion and removal, the choices offered to women who live in resource-poor settings can be limited, as they might not be able to access healthcare services when needed (91). A qualitative study in Johannesburg, South Africa, which involved interviews with healthcare professionals in HIV and primary care clinics, found that women often lack access to proper counselling and a variety of preferred methods because of staff shortages, a lack of training, and a restricted supply of contraceptives. Additionally, it was found that healthcare providers perceived injectables as the “best” contraception method for all women (110). The lack of measures of choice coupled with the training of healthcare providers to prioritise one form of contraception over the other may have a detrimental effect on women's choice.

Regarding target populations, we found that FP interventions included in our review mostly target women, specifically those accessing healthcare services for postpartum follow-up or other gynaecological issues like genital fistulas. However, truly rights-based and equitable FP services would reach vulnerable populations, who may have unique SRH needs and/or face barriers when accessing FP services. These groups include adolescents, LGBTQ+ populations, people with disabilities, and women experiencing intimate partner violence (IPV) (33, 111–113). However, none of the studies we reviewed considered or discussed the specific needs of vulnerable groups accessing FP services. In addition, the studies reviewed rarely targeted unmarried women, adolescents, or those outside of clinical settings. Targeting populations within clinical settings is a well-known public health strategy (80–82), however, similar opportunistic approaches (where people are not purposefully invited to a clinic but are reached out as they spontaneously visited it) can be effective, but may miss other vulnerable groups that may not be regularly visiting healthcare settings (54, 114–116).

Men were rarely targeted or consulted within the FP interventions we reviewed. Instead, the two studies that targeted men did so as a form of demand generation, aiming to change social norms and have men champion women's right to FP services. The wider literature is divided on whether to target men within FP programmes since men can limit or increase women's SRH autonomy within patriarchal societies (12, 113, 117–120). Some researchers propose that engaging men when it is safe can support women in the decision to use contraceptive services when and if they choose to (117, 121, 122). Others cite the association between IPV and reproductive coercion as well as the fear that men might limit women's reproductive choices (113, 123). Those that are in favour of excluding men from FP interventions assert that women should make their own reproductive decisions, independent of men (124). Rarely does the FP community describe men as clients with specific needs and desires that should receive individual services (12). Yet, the Sustainable Development Goals (SDGs) include success indicators tracking the number of countries with laws and regulations that guarantee full and equal access to both women and men to sexual and reproductive health care, information and education (118).

Most of the interventions described in our review did not address the structural determinants of gender inequality and their relevance to the sexual and reproductive health of women and girls. Likewise, most of the reviewed interventions did not acknowledge the importance of women's networks in influencing these women's choices and actions (and, by consequence, failed to include those networks in the interventions themselves). Other scholars have also identified the absence of a transformative approach within FP interventions that would reframe how couples make decisions around FP (7, 119). However, working within the relational space of a woman's couple, family, and community, where gender norms and expectations are constructed and internalised, is critically important to achieve sustainable shifts in the norms affecting women's access to modern forms of contraception (125–127). Interestingly, only three of the reviewed articles subsidised the cost of the consultation to FP services, however the specific mechanism was not specified. The limited number of papers on incentives may be due to our exclusion criteria which eliminated grey literature. Moreover, several studies we originally identified were either study protocols or symposium abstracts, which were also excluded from our review. However, evidence on providing incentives of performance-based funding in family planning programmes is mixed. Cole et al. (128) provide recommendations to make rights-based principles explicitly clear in such funding programmes as they vocalise worry on lack of quality, agency and equity with such interventions (128).

The principles of the FP2030 partnership, which emphasise the importance of voluntarism, choice, and autonomy within FP interventions provide an opportunity to tackle some of the ethical issues raised in this review and reclaim the importance of a rights-based approach. This new phase for the FP community also offers an opportunity to design and implement new measures of success that capture autonomy and informed choice. Furthermore, measures of success could also capture men's use of FP, to further promote the design of new forms of contraception directed at men as independent FP users. However, a growing body of literature is starting to make the case on how “contemporary forms of population control manifest in familiar and unexpected ways” (129) and how continuing to frame population control practices as something from the past is hampering the possibility to reconceptualize SRHR as a key focus in development agendas for all individuals (99). Scholars are also questioning how the prospect of economic growth of attaining the “demographic dividend” in SSA is perhaps excessively focused on family planning uptake (and women's bodies in the Global South) and is based in the past East Asian “miracle”, which may not be replicable in SSA where the context is significantly different (130, 131).

Through this review, we provide a first summary of how FP interventions were implemented in SSA between 2000 and 2020. We hope that this review will serve as a stepping stone that will inform future research, in order to better understand how interventions on the ground are affected by numerical targets and uptake-focused indicators set by international organisations. The findings of this review will be of interest to decision makers and designers of FP interventions wanting to proactively address ethical dimensions of FP. We hope that this study will also foster self-reflection among FP actors on how the right-based FP planning translates in the ground, be mindful of the ethical limitations that maybe be present in the FP interventions and how the power dynamics inherent in a FP community that is primarily headquartered in the Global North but operating in the Global South may influence. We advocate for further research to expand upon and refine our propositions, culminating in the development of a comprehensive framework for family planning (FP) organizations to adopt during the planning and implementation of interventions. Additionally, it is imperative to explore novel indicators firmly rooted in the principles of voluntarism, autonomy, and equity. Such research can allow towards more ethically sound and rights-based FP interventions and policies, facilitating the empowerment of individuals and fostering an inclusive and equitable approach to reproductive health initiatives.

This scoping review has several limitations. First, we only included studies written in English, which may have limited the number of studies included in our review, especially French studies on interventions based in Francophone countries in SSA. Second, we limited our search to major bibliographic databases and may have missed important and relevant studies that would have informed our results. Third, we used the terms FP and contraception interchangeably and limited our search to interventions that utilise demand generation approaches or focus on addressing unmet needs. This scoping review was also limited to FP interventions that have been peer-reviewed and published. Therefore, we may have failed to capture grey literature describing FP interventions and it is likely that this may have omitted the inclusion of all significant donors within the sector. As this review was limited to 2000–2020, further research is needed to assess any changes that may have occurred after FP2020 regarding indicators used, populations targeted, as well as new FP interventions in SSA.

## 5. Conclusion

This review demonstrates that ethical challenges in family planning programming are not an issue of the past but continue to persist today. Our examination of how family planning interventions were designed, who they target, and how they were measured from 2000 to 2020 highlights key ways in which interventions do not necessarily fulfil the reproductive and contraceptive intentions of all people. These include a focus on measuring success with uptake of modern contraception methods, an emphasis on provisioning LARCs, and married women.

The FP community must begin a transparent discussion about these ethical challenges and begin to address them in order to meet the FP2030 goals of autonomy, voluntarism, and informed choice. We hope that this review will help members of the international family planning community to reflect on ethical considerations in how their interventions are currently designed and how they might be improved. In a broader sense, we hope to demonstrate that ethical issues with FP programming are still an issue today and need to be addressed.

## Data Availability

The original contributions presented in the study are included in the article/**Supplementary Material**, further inquiries can be directed to the corresponding author.
